# Evaluation of Amyloid Polypeptide Aggregation Inhibition and Disaggregation Activity of A-Type Procyanidins

**DOI:** 10.3390/ph14111118

**Published:** 2021-10-31

**Authors:** Taisei Tanaka, Vipul V. Betkekar, Ken Ohmori, Keisuke Suzuki, Hideyuki Shigemori

**Affiliations:** 1 Graduate School of Science and Technology, University of Tsukuba, 1-1-1 Tennodai, Tsukuba 305-8572, Japan; s2121024@s.tsukuba.ac.jp; 2 Department of Chemistry, Tokyo Institute of Technology, 2-12-1 O-okayama, Meguro-ku, Tokyo 152-8551, Japan; betkekar.v.aa@m.titech.ac.jp (V.V.B.); kohmori@chem.titech.ac.jp (K.O.); ksuzuki@chem.titech.ac.jp (K.S.); 3 Faculty of Life and Environmental Sciences, University of Tsukuba, 1-1-1 Tennodai, Tsukuba 305-8572, Japan; 4 Microbiology Research Center for Sustainability (MiCS), University of Tsukuba, 1-1-1 Tennodai, Tsukuba 305-8572, Japan

**Keywords:** Alzheimer’s disease, amyloid β, A-type procyanidin, catechol, human islet amyloid polypeptide, type 2 diabetes

## Abstract

The number of people worldwide suffering from Alzheimer’s disease (AD) and type 2 diabetes (T2D) is on the rise. Amyloid polypeptides are thought to be associated with the onset of both diseases. Amyloid-β (Aβ) that aggregates in the brain and human islet amyloid polypeptide (hIAPP) that aggregates in the pancreas are considered cytotoxic and the cause of the development of AD and T2D, respectively. Thus, inhibiting amyloid polypeptide aggregation and disaggregation existing amyloid aggregates are promising approaches in the therapy and prevention against both diseases. Therefore, in this research, we evaluated the Aβ/hIAPP anti-aggregation and disaggregation activities of A-type procyanidins **1**–**7** and their substructures **8** and **9**, by conducting structure–activity relationship studies and identified the active site. The thioflavin-T (Th-T) assay, which quantifies the degree of aggregation of amyloid polypeptides based on fluorescence intensity, and transmission electron microscopy (TEM), employed to directly observe amyloid polypeptides, were used to evaluate the activity. The results showed that catechol-containing compounds **1**–**6** exhibited Aβ/hIAPP anti-aggregation and disaggregation activities, while compound **7**, without catechol, showed no activity. This suggests that the presence of catechol is important for both activities. Daily intake of foods containing A-type procyanidins may be effective in the prevention and treatment of both diseases.

## 1. Introduction

The number of people worldwide suffering from Alzheimer’s disease (AD) and type 2 diabetes (T2D) is on the rise, posing serious health problems in aging societies. Numerous studies have shown that a relationship exists between AD and T2D [1]. AD and T2D share many common pathophysiological features, including aggregation of amyloid polypeptides with an intermolecular β-sheet structure and increased oxidative stress [2,3,4]. Amyloid β (Aβ) and human islet amyloid polypeptide (hIAPP) are amyloid polypeptides responsible for AD and T2D, respectively [5,6,7]. hIAPP consists of 37 amino acids, and is secreted from pancreatic β cells, while Aβ consists of 36–43 amino acids and is produced from amyloid precursor protein in the brain [8]. Aβ and hIAPP show sequence identify (25%) and similarity (50%) [9]; both amyloid polypeptides aggregate through a similar structure called cross-β-sheet structures via the nucleation-elongation phase [10]. However, the secondary structures distributions of Aβ and hIAPP are different [11]. These aggregates attack cells in various ways [12], causing atrophy of the cerebrum and hippocampus in the brain and insulin deficiency in the pancreas. Furthermore, recent studies have shown that hIAPP is mixed in senile plaques, aggregates of Aβ, present in the brains of AD patients [13]. On the other hand, Aβ has been found to aggregate in the pancreas of transgenic mice expressing both Aβ and hIAPP [14]. Therefore, compounds that can inhibit the aggregation of both amyloid proteins would be effective drugs for the prevention and treatment of both diseases. It has been reported that epigallocatechin gallate and resveratrol display Aβ/hIAPP aggregation inhibitory activities, and considerable attention has been devoted toward polyphenols, which are abundant in various foods [15,16,17,18].

It is well known that several natural compounds, including polyphenols, can control hIAPP aggregation, and many such polyphenols have antioxidant activity, and while the hydrophobic and aromatic properties of polyphenols inhibit the formation and elongation of amyloid fibrils, their antioxidant capacity has been found to promote the destabilization of fibril aggregates [19]. Moreover, it has long been suggested that catechol is involved in the inhibition of Aβ aggregation, and a recent structure–activity relationship study using three tyrosol ligands also showed aggregation-inhibiting activity with catechol, which was attributed to the stabilization of the Aβ-ligand interaction by H-bonding to Glu22 by the hydroxyl group of catechol [20].

**Figure 1:**
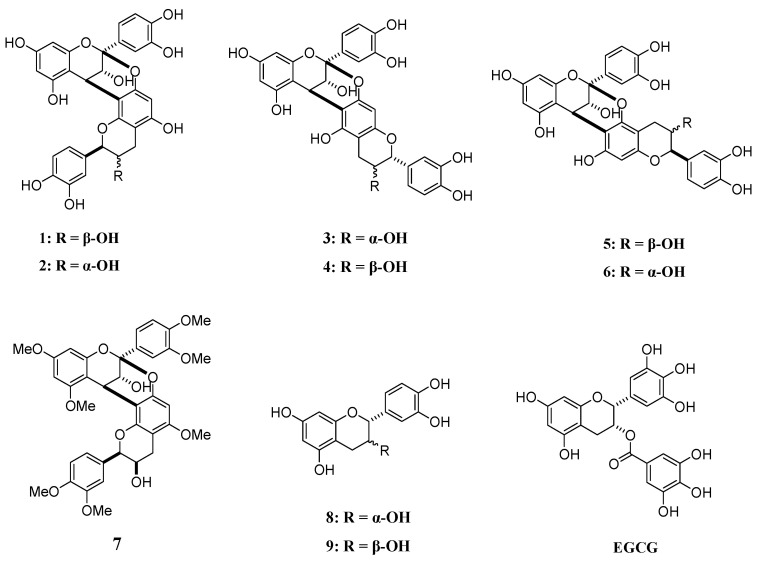
Structural formulae of compounds **1–9** and EGCG.

We have previously reported that caffeoylquinic acid, phenylethanoid glycoside, and hispidin derivatives inhibit Aβ42 aggregation [21,22,23,24]. We have also recently shown that kukoamines A and B, schizotenuin A, lycopic acids, rosmarinic acid, and clovamide exhibit inhibitory activity against Aβ/hIAPP aggregation [25,26,27,28,29]. These compounds, which inhibit Aβ42/hIAPP aggregation, all contain a catechol moiety, and catechol-type polyphenols can potentially inhibit amyloid protein aggregation. In this study, we focused on A-type procyanidins, which have two catechols, and investigated their effects on the aggregation of amyloid proteins. A-type procyanidins are found in peanut skin and consist of (+)-catechin or (−)-epicatechin. In addition, to identify the active site, anti-aggregation activity tests of Aβ42/hIAPP were performed and structure–activity correlations were examined by use of A-type procyanidins **1**–**7** and their substructures **8** and **9** (Figure 1). Furthermore, degradation of already aggregated amyloid polypeptides (disaggregation activity) was also evaluated, as well as the antioxidant activity of these compounds.

## 2. Results

### 2.1. Evaluation of Aβ42 Aggregation Inhibitory Activity of Compounds ***1**–**9***

**Figure 2:**
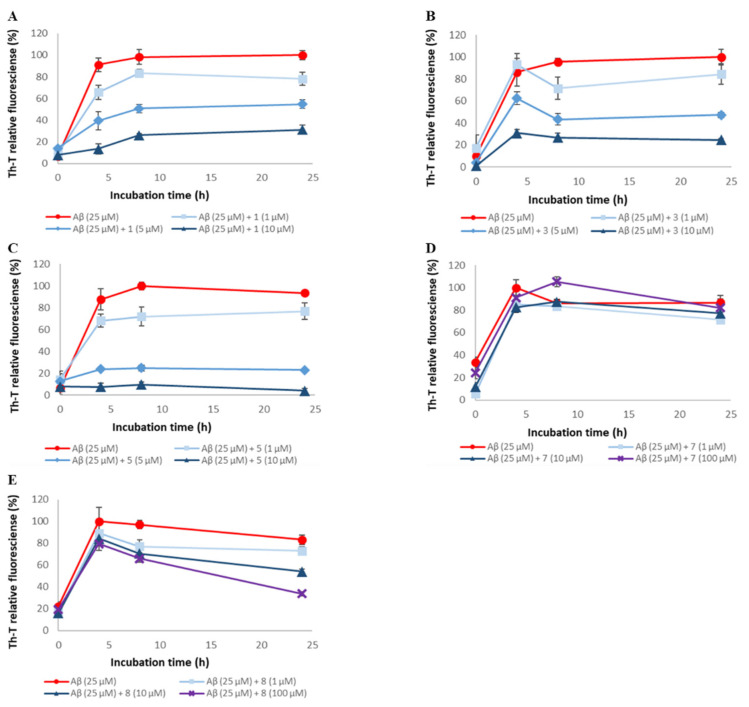
Efficacy of compounds **1**, **3**, **5**, **7**, and **8** against Aβ42 aggregation. Aβ42 (25 μM) fibril formation was monitored by Th-T fluorescence with varying concentrations of these compounds. (**A**) **1**, (**B**) **3**, (**C**) **5**, (**D**) **7**, and (**E**) **8**. At an excitation wavelength of 420 nm and an emission wavelength of 485 nm, fluorescence intensity was measured. Each value is represented the mean ± SD (*n* = 6).

To assess the ability of synthetic A-type procyanidins **1**–**7** [30] and their substructures **8** and **9** to inhibit Aβ42 aggregation, thioflavin-T (Th-T) assay was conducted (Figure 2 and Figure S1). In this study, (−)-epigallocatechin gallate (EGCG), the activity of which has been reported in previous studies, was used as the positive control [15,17,31]. The IC₅₀ values for these compounds are shown in Table 1. Aβ42 aggregation was inhibited in a concentration-dependent manner by all the compounds except compound **7**. The Aβ42 aggregation inhibitory activity of these compounds was as follows: **1**, **2**, **3**, **4**, **5**, and **6** > **8** and **9** >> **7**. These results suggest that compounds containing two catechols are more active than those with one (the activity is proportional to the number of catechols) and that the presence of catechol is important.

**Figure 3:**
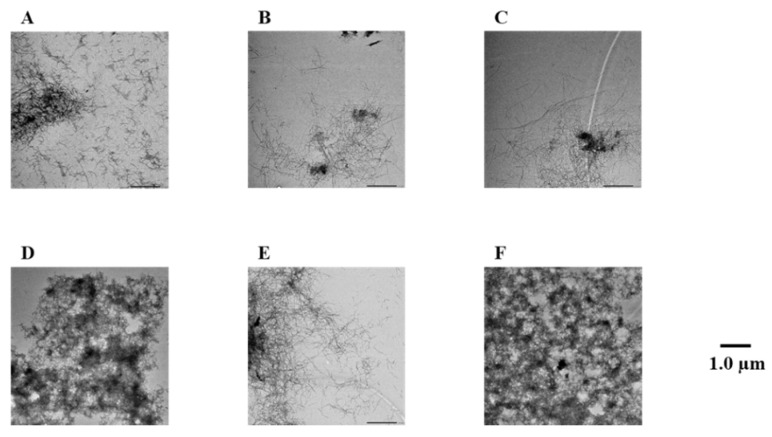
Efficacy of compounds **1**, **3**, **5**, **7**, and **8** against Aβ42 fibril formation visualized by use of TEM. The formation of Aβ42 fibrils was observed after incubation in 50 µM PBS buffer for 24 h. Scale bar: 1.0 µM. (**A**) Aβ42 + **1**, (**B**) Aβ42 + **3**, (**C**) Aβ42 + **5**, (**D**) Aβ42 + **7**, (**E**) Aβ42 + **8**, and (**F**) Aβ42.

To confirm the results of the Th-T assay, Aβ42 fibrils were observed directly using TEM (Figure 3 and Figure S2). In the case of the Aβ42-only reaction solution (no compound added), it was confirmed that many Aβ42 aggregates were spread out in a mesh-like pattern. Similar results were obtained for compound **7**, which showed no activity in the Th-T assay. By contrast, compounds **1**–**6**, **8**, and **9**, which showed activity in the Th-T assay, gave rise to reduced aggregation compared to Aβ42 alone. Furthermore, these results show that compounds with two catechols are more active than those with one. These results support the results of the Th-T assay.

### 2.2. Evaluation of hIAPP Aggregation Inhibitory Activity of Compounds ***1**–**9***

**Figure 4:**
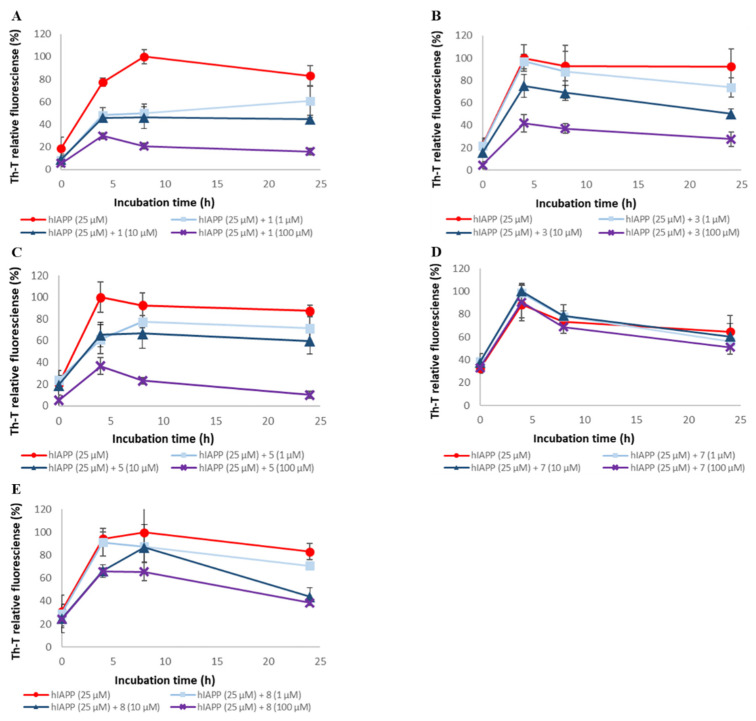
Efficacy of compounds **1**, **3**, **5**, **7**, and **8** against hIAPP aggregation. hIAPP (25 μM) fibril formation was monitored by Th-T fluorescence with varying concentrations of these compounds. (**A**) **1**, (**B**) **3**, (**C**) **5**, (**D**) **7**, and (**E**) **8**. At an excitation wavelength of 420 nm and an emission wavelength of 485 nm, fluorescence intensity was measured. Each value is represented the mean ± SD (*n* = 6).

To assess the ability of synthetic A-type procyanidins **1–7** and their substructures **8** and **9** to inhibit hIAPP aggregation, thioflavin-T (Th-T) assay was conducted (Figure 4 and Figure S3). The IC₅₀ values for these compounds are shown in Table 1. hIAPP aggregation was inhibited in a concentration-dependent manner by all the compounds except compound **7**. The hIAPP aggregation inhibitory activities of these compounds were as follows: **1**, **2**, **3**, **4**, **5**, and **6** > **8** and **9** >> **7**. These results suggest that the presence of catechol is important for activity and that the activity increases in proportion to the number of catechols. The hIAPP aggregation inhibitory activity of each compound was higher than its Aβ42 aggregation inhibitory activity, but the overall trend was similar to that of the Aβ42 aggregation inhibitory activity.

**Figure 5:**
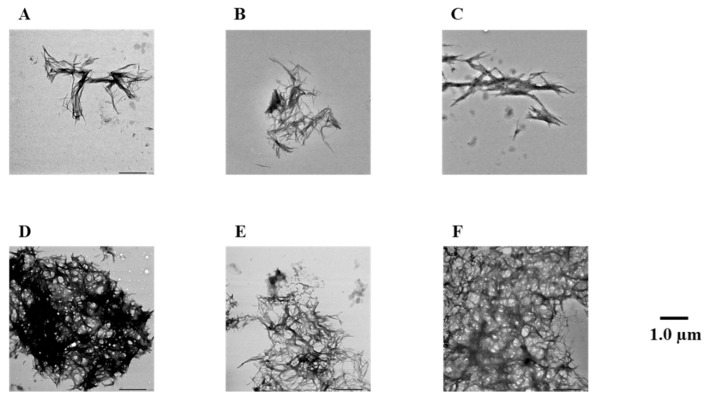
Efficacy of compounds **1**, **3**, **5**, **7**, and **8** against hIAPP fibril formation visualized by use of TEM. The formation of hIAPP fibrils was observed after incubation in 50 µM PBS buffer for 24 h. Scale bar: 1.0 µM. (**A**) hIAPP + **1**, (**B**) hIAPP + **3**, (**C**) hIAPP + **5**, (**D**) hIAPP + **7**, (**E**) hIAPP + **8**, and (**F**) hIAPP.

To confirm the results of the Th-T assay, the hIAPP fibrils were observed directly using TEM (Figure 5 and Figure S4). In the case of the hIAPP-only reaction solution (no compound added), it was confirmed that numerous hIAPP aggregates were distributed in a mesh-like pattern. Similar results were obtained for compound **7**, which showed no activity in the Th-T assay. In contrast, compounds **1**–**6**, **8**, and **9**, which showed activity in the Th-T assay, gave rise to reduced aggregation compared to hIAPP alone. Furthermore, these results indicated that compounds with two catechols were more active than those with one. These results support the results of the Th-T assay.

### 2.3. Evaluation of Disaggregation Activity of Compounds ***1***, ***3***, ***5***, ***7***, and ***8*** on Pre-Existing Aβ42 Aggregates

**Figure 6:**
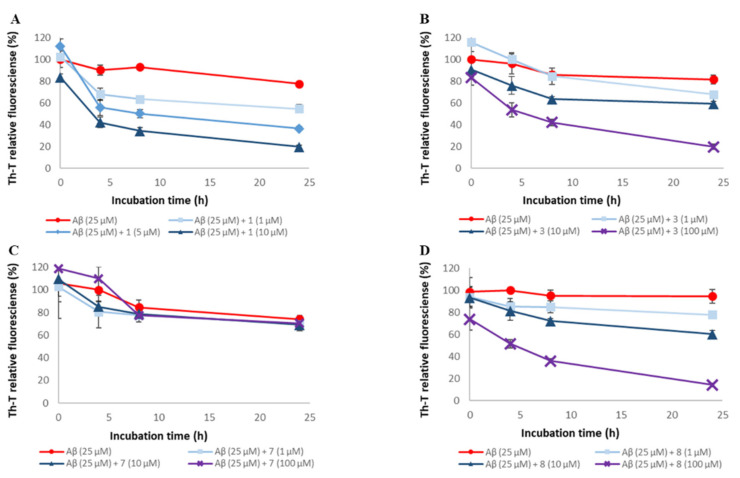
Efficacy of compounds **1**, **3**, **7,** and **8** against pre-existing Aβ42 aggregates. Aggregates of Aβ42 (25 μM) were monitored by Th-T fluorescence with varying concentrations of these compounds. (**A**) **1**, (**B**) **3**, (**C**) **7**, and (**D**) **8**. At an excitation wavelength of 420 nm and an emission wavelength of 485 nm, fluorescence intensity was measured. Each value is represented the mean ± SD (*n* = 6).

To assess the disaggregation ability of compounds **1**, **3**, **5**, **7**, and **8** on Aβ42 aggregates, thioflavin-T (Th-T) assay was conducted (Figure 6 and Figure S5). These compounds were selected based on the number of catechols they contained, their steric structure, and constituent units. The EC₅₀ values for these compounds are shown in Table 2. For all the compounds except compound **7**, Aβ42 aggregates were disaggregated concentration-dependently. The disaggregation activities of these compounds on Aβ42 aggregates were as follows: **1** and **5** > **3** and **8** >> **7**. These results suggest that the presence of catechol is important for Aβ42 disaggregation activity. On the other hand, Aβ42 disaggregation activities showed a different trend from the aggregation inhibition activities, as there was a difference in activity even when the number of catechols was the same.

**Figure 7:**
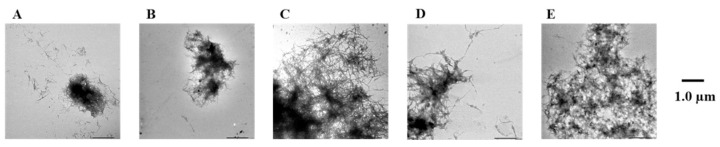
Efficacy of compounds **1**, **3**, **7**, and **8** against pre-existing Aβ42 aggregates visualized by use of TEM. Scale bars: 1.0 µm. (**A**) Aβ42 + **1**, (**B**) Aβ42 + **3**, (**C**) Aβ42 + **7**, (**D**) Aβ42 + **8**, and (**E**) Aβ42.

To confirm the results of the Th-T assay, the Aβ42 fibrils were observed directly using TEM (Figure 7 and Figure S6). In the case of the Aβ42-only reaction solution (no compound added), the presence of copious aggregates of Aβ42 distributed in a mesh-like pattern was confirmed. Similar results were obtained for compound **7**, which showed no activity in the Th-T assay. In contrast, reduced aggregation was noted in the presence of compounds **1**, **3**, **5**, and **8**, which showed activity in the Th-T assay, compared to Aβ42 alone. These results support the results of the Th-T assay.

### 2.4. Evaluation of Disaggregation Activity of Compounds ***1***, ***3***, ***5***, ***7***, and ***8*** on Pre-Existing hIAPP Aggregates

**Figure 8:**
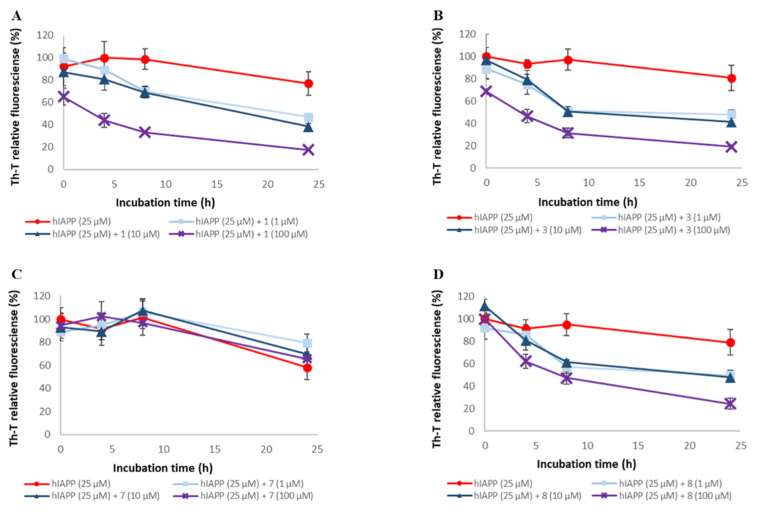
Efficacy of compounds **1**, **3**, **7**, and **8** against pre-existing hIAPP aggregates. Aggregates of hIAPP (25 μM) were monitored by Th-T fluorescence with varying concentrations of these compounds. (**A**) **1**, (**B**) **3**, (**C**) **7**, and (**D**) **8**. At an excitation wavelength of 420 nm and an emission wavelength of 485 nm, fluorescence intensity was measured. Each value is represented the mean ± SD (*n* = 6).

To assess the disaggregation ability of compounds **1**, **3**, **5**, **7**, and **8** on hIAPP aggregates, thioflavin-T (Th-T) assay was conducted (Figure 8 and Figure S7). The EC₅₀ values for these compounds are shown in Table 2. For all the compounds except compound **7**, hIAPP aggregates were disaggregated concentration-dependently. The disaggregation activities of these compounds on hIAPP aggregates were as follows: **1** and **3** > **5** and **8** >> **7**. Therefore, it suggests the importance of the presence of catechol for the disaggregation of hIAPP. Moreover, hIAPP disaggregation activity showed a different trend from the aggregation inhibition activity, as there was a difference in activity even when the number of catechols was the same. This trend was different from that of hIAPP aggregation inhibitory activity.

**Figure 9:**
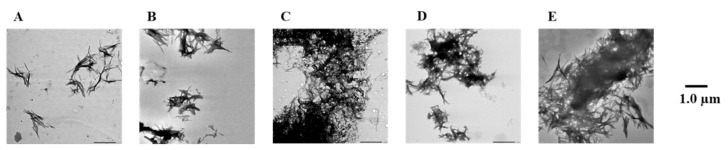
Efficacy of compounds **1**, **3**, **7**, and **8** against pre-existing hIAPP aggregates visualized by use of TEM. Scale bars: 1.0 µm. (**A**) hIAPP + **1**, (**B**) hIAPP + **3**, (**C**) hIAPP + **7**, (**D**) hIAPP + **8**, and (**E**) hIAPP.

To confirm the results of the Th-T assay, the hIAPP fibrils were observed directly using TEM (Figure 9 and Figure S8). In the case of the hIAPP-only reaction solution (no compound added), the presence of numerous aggregates of Aβ distributed in a mesh-like pattern was observed. Similar results were obtained for compound **7**, which showed no activity in the Th-T assay. In contrast, in the presence of compounds **1**, **3**, **5**, and **8**, which showed activity in the Th-T assay, aggregation was reduced compared to hIAPP alone. These results support the results of the Th-T assay.

### 2.5. Evaluation of Antioxidant Activity of A-Type Procyanidins and Their Related Compounds

To assess the antioxidant potential of the compounds **1**–**9**, 2,2-diphenyl-1-picrylhydrazyl (DPPH) free-radical-scavenging assay was conducted. The IC₅₀ values for these compounds are shown in Table 3. All of the compounds, except compound **7,** exhibited radical-scavenging activity, which increased in a concentration-dependent manner and show high antioxidant rate at concentration of 50 μM. These results suggest that the presence of phenolic hydroxyl groups is important for radical scavenging activity. In addition, the antioxidant activities among the A-type procyanidins were comparable.

## 3. Discussion

In this research, we examined the effects of compounds **1**–**9** on the aggregation and disaggregation of Aβ42 and hIAPP, as well as their antioxidant properties.

The results of structure–activity relationship studies of A-type procyanidin derivatives confirmed that catechol is important for the inhibition of Aβ42 aggregation. In addition, compounds bearing two catechols were more active than those with one catechol. This trend is consistent with previous research showing that polyphenols with multiple catechol moieties exhibit higher Aβ42 aggregation inhibitory activities [21,22,23,24,25,26,27,28]. In addition, it was surmised that the steric structure and constituent units did not have a significant effect on the activity. Catechol was also important for hIAPP aggregation inhibitory activity, which followed a similar trend to that of Aβ42 aggregation inhibitory activity.

This tendency is consistent with the results of previous studies [25,26,27,29]. However, the IC₅₀ values for hIAPP aggregation were higher than those for Aβ42 aggregation. This may be due to the differences in the amino acid sequence and 3D structure between Aβ42 and hIAPP, which affects their affinity for the compounds.

The catechol moiety readily auto-oxidizes to form *o*-benzoquinone, which may covalently bind to nucleophilic amino acid residues of amyloid proteins (Michael addition and Schiff base formation) and destabilize the β-sheet structure [32,33,34]. The fact that the activity increased in proportion to the number of catechols is thought to be due to this mechanism. On the other hand, compound **7** is suggested to have no amyloid polypeptide aggregation inhibitory activity because *o*-benzoquinone is not formed in compound **7**. Previous research has indicated that π–π stacking interactions between amino acid residues of Aβ42 and the aromatic ring of the compounds acting as an inhibitor as well as hydrogen bond are possible factors that govern the inhibition of Aβ42 β-sheet formation [35]. However, compound **7**, which has four aromatic rings and no phenolic hydroxyl group, did not show amyloid polypeptide aggregation inhibitory activity, suggesting that π–π stacking interactions may not be involved in the amyloid polypeptide aggregation inhibition mechanism of A-type procyanidin derivatives. On the other hand, because the bulkiness of the methyl group of **7** may prevent it from entering the space between amino acid residues, we plan to examine the inhibitory activity of A-type procyanidins on amylolytic polypeptide aggregation under conditions where catechol is not oxidized by adding a reducing agent. A more detailed analysis will be conducted in the future.

For Aβ42/hIAPP disaggregation activity of A-type procyanidin derivatives, structure–activity relationship studies indicated that the presence of catechol is important for their activity. However, unlike the Aβ42/hIAPP aggregation inhibitory activity, the Aβ42/hIAPP disaggregation activity results suggest that the steric structure also contributes significantly to the activity. The reason for these differences is that, unlike monomers, amyloid polypeptides form aggregates and access to them is restricted. Moreover, several compounds showed different activities against each aggregate, which may be due to differences in accessibility resulting from differences in the steric structure and secondary structure distribution of Aβ aggregates and hIAPP aggregates. Catechin and epicatechin have been reported to destabilize Aβ fibrils, and several other aromatic compounds have been reported to degrade amyloid polypeptide fibrils. However, the disaggregation mechanism remains unclear; therefore, it is necessary to clarify this mechanism in the future.

The results of the DPPH radical scavenging activity test confirmed the antioxidant activity of all the A-type procyanidin compounds except compound **7**, suggesting the importance of phenolic hydroxyl groups. It has been reported that amyloid polypeptides generate radicals during the aggregation process, leading to further aggregation and cell death [36,37,38]. On the other hand, as the DPPH radical does not exist in the body, antioxidant activity must be further evaluated from additional perspectives, such as the superoxide dismutase (SOD) activity test.

It has been reported that procyanidins with a low degree of polymerization, such as dimers, can penetrate the blood-brain barrier (BBB). All procyanidins used in this study were dimers. Therefore, in this research, we investigated A-type procyanidins for their Aβ42/hIAPP aggregation inhibitory, Aβ42/hIAPP disaggregation, and antioxidant activities, and showed that these active compounds have significant potential for use as preventive and therapeutic agents for both diseases. On the other hand, nobiletin, an *O*-methoxylated flavonoid, has been reported to have the potential to cause demethylation in vivo [39] and to show efficacy in AD model mice [40]. Therefore, although compound **7** did not show any activity in the in vitro experimental system conducted in this study, the presence or absence of in vivo activity needs to be investigated in the future. The results of this research may contribute to the development of preventive and therapeutic agents for AD and T2D. In the future, we aim to elucidate the inhibitory and disaggregation mechanisms of A-type procyanidins in more detail. Furthermore, it is necessary to investigate the cytoprotective activity using cells and the preventive effect on cognitive function using mice as an in vivo experiment.

The results of this research suggest that dietary materials containing high amounts of A-type procyanidins can potentially contribute to the development of functional foods for the prevention of AD and T2D.

## 4. Materials and Methods

### 4.1. A-Type Procyanidins and Their Substructures

Compounds **1**–**7** used in this study were synthesized [30], while compounds **8**, **9**, and epigallocatechin gallate (EGCG) were purchased from Merck (Figure 1).

### 4.2. Thioflavin T (Th-T) Assay

The degree of aggregation of Aβ42/hIAPP was assessed using the Th-T method developed by Naiki et al. [41]. The procedure for this is described elsewhere [42]. Briefly, hIAPP (KareBay Biochem Inc., Monmouth Junction, NJ, USA) was dissolved in HFIP solution (1% acetic acid aqueous solution = 1:1), and Aβ42 was dissolved in 0.1% NH_4_OH solution at 250 µM. The amyloid solution was diluted tenfold with 50 mM PBS (pH 7.4) and incubated with or without samples. The peptide solution (2.5 μL) was added to 250 μL of 1 mM Th-T in 50 mM Gly-NaOH (pH = 8.5). Using a Wallac 1420 ARVO MX Multidetection Microplate Reader (PerkinElmer), the fluorescence intensity was measured at an excitation wavelength of 420 nm and an emission wavelength of 485 nm, and IC_50_ values of each compound were calculated based on the percentage inhibition of amyloid polypeptide aggregation (%) after incubation at 37 °C for 24 h. In the disaggregation activity test, amyloid polypeptides were pre-incubated for 24 h to form aggregates beforehand, and then the compounds were added. EGCG, which is known to show aggregation inhibition and disaggregation activities against amyloid polypeptide, was used as the positive control in this assay [15,17,32].

### 4.3. Transmission Electron Microscope (TEM) Observations

Aβ42 and hIAPP (25 μM each) were treated with compounds **1**–**9** and EGCG (10 μM for Aβ and 100 μM for hIAPP), dropped onto carbon-coated Formvar grids, incubated at room temperature for 2 min, washed twice with H_2_O, and air-dried for 5 min. After 24 h of incubation, the samples were observed using a JEOL JEM-1400 electron microscope.

### 4.4. DPPH Radical-Scavenging Assay

Each sample (10 μL) in MeOH was mixed with 2,2-diphenyl-1-picrylhydrazyl (DPPH) solution (1 mM in EtOH/0.4 mM 2-morpholinoethanesulfonic acid (MES) buffer (pH 6.1)/Milli Q, 4:1:3) (190 μL). It was incubated in the dark for 15 min at room temperature, and then the absorbance was measured at 490 nm (A sample). The absorbance of the negative control (A control) consisting of solvent only and blank without DPPH (A blank) were also measured at 490 nm. DPPH radical-scavenging activity was calculated by use of the following equation:

DPPH radical-scavenging activity (%) = (1 − [A sample − A blank]/A control) × 100.

## 5. Conclusions

Structure–activity relationship studies by Th-T assay and TEM observation were performed to investigate the Aβ/hIAPP anti-aggregation and disaggregation activities of A-type procyanidins **1**–**7** and their substructures **8** and **9**. These results suggested that A-type procyanidins **1**–**6** with two catechol moieties exhibited potent Aβ/hIAPP anti-aggregation and disaggregation activities, while compound **7**, without catechol, showed no activity. This suggests that the presence of catechol is important for both activities.

Therefore, this study suggests that dietary materials, containing high amounts of A-type procyanidins, may contribute to the development of functional foods for the prevention of AD and T2D.

## Figures and Tables

**Table 1:** Efficacy of compounds **1–9** against Aβ and hIAPP aggregations.

Compounds	IC_50_ (Aβ/hIAPP, µM) ^a^
**1** (procyanidin A2)	4.8/8.4
**2** (procyanidin A1)	4.6/12.9
**3** (proanthocyanidin A6)	4.2/16.4
**4**	5.7/16.8
**5** (proanthocyanidin A7)	2.4/13.4
**6**	6.8/11.6
**7**	>100/>100
**8** [(−)-epicatechin)]	41.7/38.9
**9** [(+)-catechin)]	56.8/40.9
EGCG (positive control)	3.8/1.1

^a^ IC_50_ values were calculated based on the percentage (%) inhibition of amyloid polypeptide aggregation by Th-T assay after 24 h for each compound whose concentrations was changed.

**Table 2:** Efficacy of compounds **1**, **3**, **5**, **7**, and **8** against pre-existing Aβ42 and hIAPP aggregates.

Compound	EC_50_ (Aβ/hIAPP, µM) ^a^
**1** (procyanidin A2)	3.3/5.4
**3** (proanthocyanidin A6)	23.0/4.9
**5** (proanthocyanidin A7)	8.9/15.8
**7**	>100/>100
**8** ((−)-epicatechin)	12.9/12.2
EGCG (positive control)	5.0/3.7

^a^ EC_50_ values were calculated based on the disaggregation effective rate (%) of amyloid polypeptide aggregation by Th-T assay after 24 h for each compound whose concentrations was changed.

**Table 3:** Efficacy of compounds **1**–**9** against DPPH free radical.

Compound	IC_50_ (µM) ^a^
**1** (procyanidin A2)	17.9
**2** (procyanidin A1)	12.9
**3** (proanthocyanidin A6)	14.9
**4**	14.4
**5** (proanthocyanidin A7)	14.7
**6**	14.6
**7**	>50.0
**8** [(-)-epicatechin]	18.6
**9** [(+)-catechin]	28.8
EGCG (positive control)	9.5

^a^ IC_50_ values were calculated from DPPH radical-scavenging rate (%) of each compound at varying concentration.

## Data Availability

Data is contained within the article or Supplementary Material.

## Supplementary Materials

The following are available online at https://www.mdpi.com/article/10.3390/ph14111118/s1, Figure S1: Efficacy of compounds **1**–**9** and EGCG against Aβ42 aggregation. Figure S2: Efficacy of compounds **1**–**9** and EGCG against Aβ42 fibril formation visualized by use of TEM. Figure S3: Efficacy of compounds **1**–**9** and EGCG against hIAPP aggregation. Figure S4: Efficacy of compounds **1**–**9** and EGCG against hIAPP fibril formation visualized by use of TEM. Figure S5: Efficacy of compounds **1**, **3**, **5**, **7**, **8**, and EGCG against pre-existing Aβ42 aggregates. Figure S6: Efficacy of compounds **1**, **3**, **7**, and **8** against pre-existing Aβ42 aggregates visualized by use of TEM. Figure S7: Efficacy of compounds **1**, **3**, **5**, **7**, **8**, and EGCG against pre-existing hIAPP aggregates. Figure S8: Efficacy of compounds **1**, **3**, **7**, and **8** against pre-existing hIAPP aggregates visualized by use of TEM.
