# Supplementary material for: Evaluation of Amyloid Polypeptide Aggregation Inhibition and Disaggregation Activity of A-Type Procyanidins
Source: Pharmaceuticals (Basel). 2021 Oct 31;14(11):1118. doi: 10.3390/ph14111118 (PMC8623168; doi:10.3390/ph14111118)
Supplement: Supplementary file 1 [file pharmaceuticals-14-01118-s001.zip › pharmaceuticals-1419397-supplementary.pdf]

Supplementary Materials

Evaluation of aggregation inhibition and disaggregation activity of A-type procyanidins against amyloid polypeptides

Taisei Tanaka[1]·Vipul V. Betkekar[2]·Ken Ohmori[2]·Keisuke Suzuki[2]·Hideyuki Shigemori[3,4*]

[1]Graduate School of Life and Environmental Sciences, University of Tsukuba, 1-1-1 Tennodai, Tsukuba, Ibaraki 305-8572, Japan

[2]Department of Chemistry, Tokyo Institute of Technology, 2-12-1, O-okayama, Meguroku, Tokyo 152-8551, Japan.

[3]Faculty of Life and Environmental Sciences, University of Tsukuba, 1-1-1 Tennodai, Tsukuba, Ibaraki 305-8572, Japan

[4]Microbiology Research Center for Sustainability (MiCS), University of Tsukuba, Tsukuba, Ibaraki 305-8572, Japan

E-mail: shigemori.hideyuk.fn@u.tsukuba.ac.jp

Tel: +81-29-853-4603

ORCID: 0000-0001-9778-8057

Figure S1. Efficacy of compounds **1-9** and EGCG against Aβ42 aggregation. The fibril formation of Aβ42 (25 μM) was monitored by Th-T fluorescence and the presence of A: **1** (Procyanidin A2), B: **2** (Procyanidin A1), C: **3** (Proanthocyanidin A6), D: **4**, E: **5** (Proanthocyanidin A7), F: **6**, G: **7** (Me-Procyanidin A2), H: **8** ((-)-Epicatechin), I: **9** ((+)-Catechin) and J: EGCG. Fluorescence intensity was measured at an excitation wavelength of 420 nm and emission wavelength of 485 nm. Values represent the mean ± SD (n = 6).

Figure S2. Efficacy of compounds **1-9** and EGCG against Aβ42 fibrillogenesis visualized by use of TEM. Fibril formation was observed after 24 h of incubation in 50 μM PBS buffer. Scale bars: 1.0 μm. A: Aβ42 + **1** (Procyanidin A2), B: Aβ42 + **2** (Procyanidin A1), C: Aβ42 + **3** (Proanthocyanidin A6), D: Aβ42 + **4**, E: Aβ42 + **5** (Proanthocyanidin A7), F: Aβ42 + **6**, G: Aβ42 + **7** (Me-Procyanidin A2), H: Aβ42 + **8** ((-)-Epicatechin), I: Aβ42 + **9** ((+)-Catechin), J: Aβ42 + EGCG and K: Aβ42.

Figure S3. Efficacy of compounds **1-9** and EGCG against hIAPP aggregation. The fibril formation of hIAPP (25 μM) was monitored by Th-T fluorescence and the presence of A: **1** (Procyanidin A2), B: **2** (Procyanidin A1), C: **3** (Proanthocyanidin A6), D: **4**, E: **5** (Proanthocyanidin A7), F: **6**, G: **7** (Me-Procyanidin A2), H: **8** ((-)-Epicatechin), I: **9** ((+)-Catechin) and J: EGCG. Fluorescence intensity was measured at an excitation wavelength of 420 nm and emission wavelength of 485 nm. Values represent the mean ± SD (n = 6).

Figure S4. Efficacy of compounds **1-9** and EGCG against hIAPP fibrillogenesis visualized by use of TEM. Fibril formation was observed after 24 h of incubation in 50 μM PBS buffer. Scale bars: 1.0 μm. A: hIAPP + **1** (Procyanidin A2), B: hIAPP + **2** (Procyanidin A1), C: hIAPP + **3** (Proanthocyanidin A6), D: hIAPP + **4**, E: hIAPP + **5** (Proanthocyanidin A7), F: hIAPP + **6**, G: hIAPP + **7** (Me-Procyanidin A2), H: hIAPP + **8** ((-)-Epicatechin), I: hIAPP + **9** ((+)-Catechin), J: hIAPP + EGCG and K: hIAPP.

Figure S5. Efficacy of compounds **1**, **3**, **5**, **7**, **8**, and EGCG against pre-existing Aβ42 fibrils. Aβ42 (25 μM) fibrils was quantified by monitoring Th-T fluorescence in the presence of varying concentrations of A: **1** (Procyanidin A2), B: **3** (Proanthocyanidin A6), C: **5** (Proanthocyanidin A7), D: **7** (Me-Procyanidin A2), E: **8** ((-)-Epicatechin) and F: EGCG. Fluorescence intensity was measured at an excitation wavelength of 420 nm

and emission wavelength of 485 nm. Values represent the mean ± SD (n = 6).

Figure S6. Efficacy of compounds **1**, **3**, **7**, and **8** against pre-existing Aβ42 fibrils visualized by use of TEM. Scale bars: 1.0 µm. A: Aβ42 + **1** (Procyanidin A2), B: Aβ42 + **3** (Proanthocyanidin A6), C: Aβ42 + **5** (Proanthocyanidin A7), D: Aβ42 + **7** (Me-Procyanidin A2), E: Aβ42 + **8** ((-)-Epicatechin), F: Aβ42 + EGCG and G: Aβ42.

Figure S7. Efficacy of compounds **1**, **3**, **5**, **7**, **8** and EGCG against pre-existing hIAPP fibrils. hIAPP (25 µM) fibrils was quantified by monitoring Th-T fluorescence in the presence of varying concentrations of A: **1** (Procyanidin A2), B: **3** (Proanthocyanidin A6), C: **5** (Proanthocyanidin A7), D: **7** (Me-Procyanidin A2), E: **8** ((-)-Epicatechin) and F: EGCG. Fluorescence intensity was measured at an excitation wavelength of 420 nm and emission wavelength of 485 nm. Values represent the mean ± SD (n = 6).

Figure S8. Efficacy of compounds **1**, **3**, **7**, and **8** against pre-existing hIAPP fibrils visualized by use of TEM. Scale bars: 1.0 µm. A: hIAPP + **1** (Procyanidin A2), B: hIAPP + **3** (Proanthocyanidin A6), C: hIAPP + **5** (Proanthocyanidin A7), D: hIAPP + **7** (Me-Procyanidin A2), E: hIAPP + **8** ((-)-Epicatechin), F: hIAPP + EGCG and G: hIAPP.

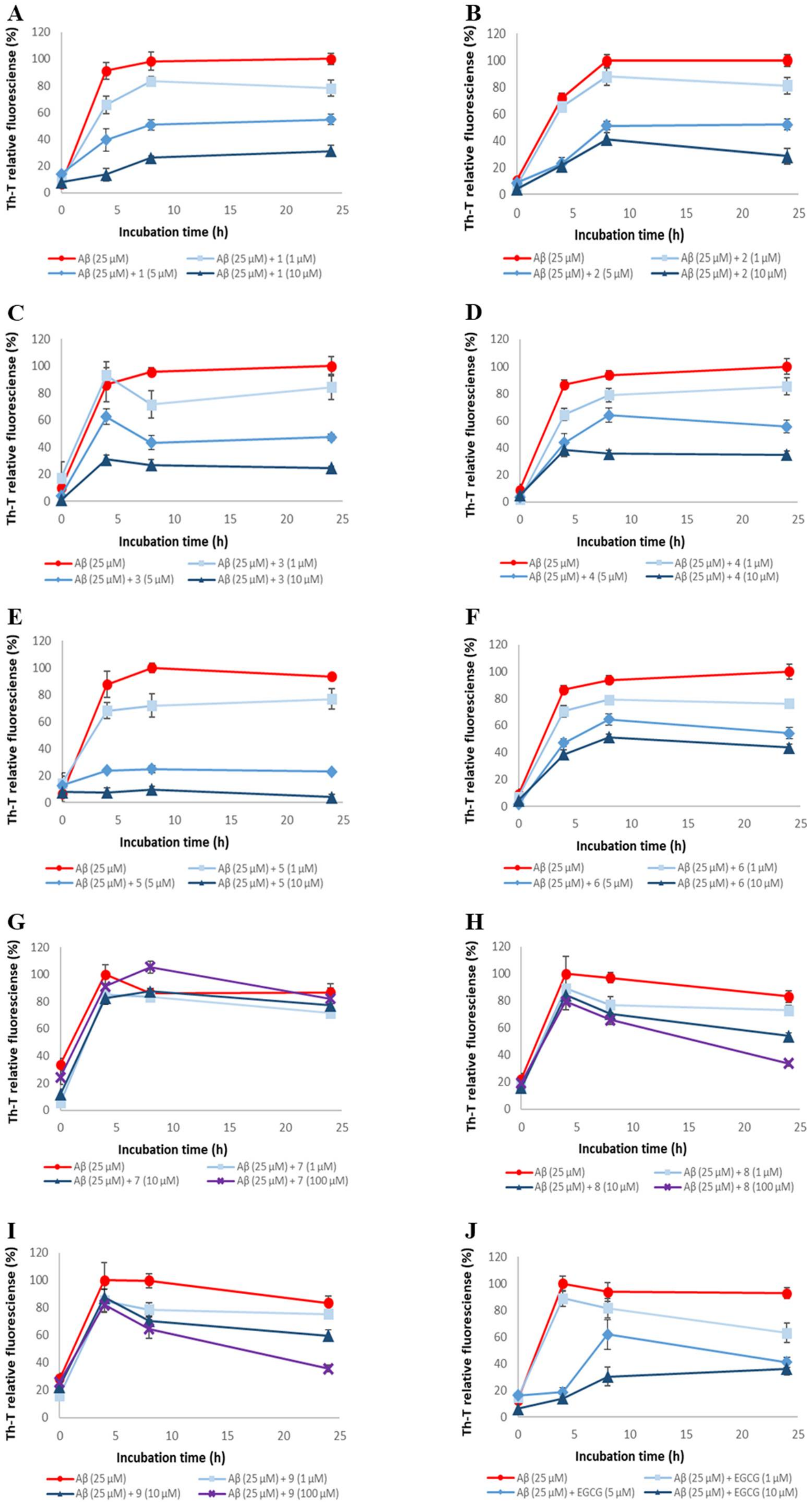
A
Th-T relative fluoresciense (%)
Incubation time (h)
Aβ (25 µM)
Aβ (25 µM) + 1 (1 µM)
Aβ (25 µM) + 1 (5 µM)
Aβ (25 µM) + 1 (10 µM)
B
Th-T relative fluoresciense (%)
Incubation time (h)
Aβ (25 µM)
Aβ (25 µM) + 2 (1 µM)
Aβ (25 µM) + 2 (5 µM)
Aβ (25 µM) + 2 (10 µM)
C
Th-T relative fluoresciense (%)
Incubation time (h)
Aβ (25 µM)
Aβ (25 µM) + 3 (1 µM)
Aβ (25 µM) + 3 (5 µM)
Aβ (25 µM) + 3 (10 µM)
D
Th-T relative fluoresciense (%)
Incubation time (h)
Aβ (25 µM)
Aβ (25 µM) + 4 (1 µM)
Aβ (25 µM) + 4 (5 µM)
Aβ (25 µM) + 4 (10 µM)
E
Th-T relative fluoresciense (%)
Incubation time (h)
Aβ (25 µM)
Aβ (25 µM) + 5 (1 µM)
Aβ (25 µM) + 5 (5 µM)
Aβ (25 µM) + 5 (10 µM)
F
Th-T relative fluoresciense (%)
Incubation time (h)
Aβ (25 µM)
Aβ (25 µM) + 6 (1 µM)
Aβ (25 µM) + 6 (5 µM)
Aβ (25 µM) + 6 (10 µM)
G
Th-T relative fluoresciense (%)
Incubation time (h)
Aβ (25 µM)
Aβ (25 µM) + 7 (1 µM)
Aβ (25 µM) + 7 (10 µM)
Aβ (25 µM) + 7 (100 µM)
H
Th-T relative fluoresciense (%)
Incubation time (h)
Aβ (25 µM)
Aβ (25 µM) + 8 (1 µM)
Aβ (25 µM) + 8 (10 µM)
Aβ (25 µM) + 8 (100 µM)
I
Th-T relative fluoresciense (%)
Incubation time (h)
Aβ (25 µM)
Aβ (25 µM) + 9 (1 µM)
Aβ (25 µM) + 9 (10 µM)
Aβ (25 µM) + 9 (100 µM)
J
Th-T relative fluoresciense (%)
Incubation time (h)
Aβ (25 µM)
Aβ (25 µM) + EGCG (1 µM)
Aβ (25 µM) + EGCG (5 µM)
Aβ (25 µM) + EGCG (10 µM)

Figure S1

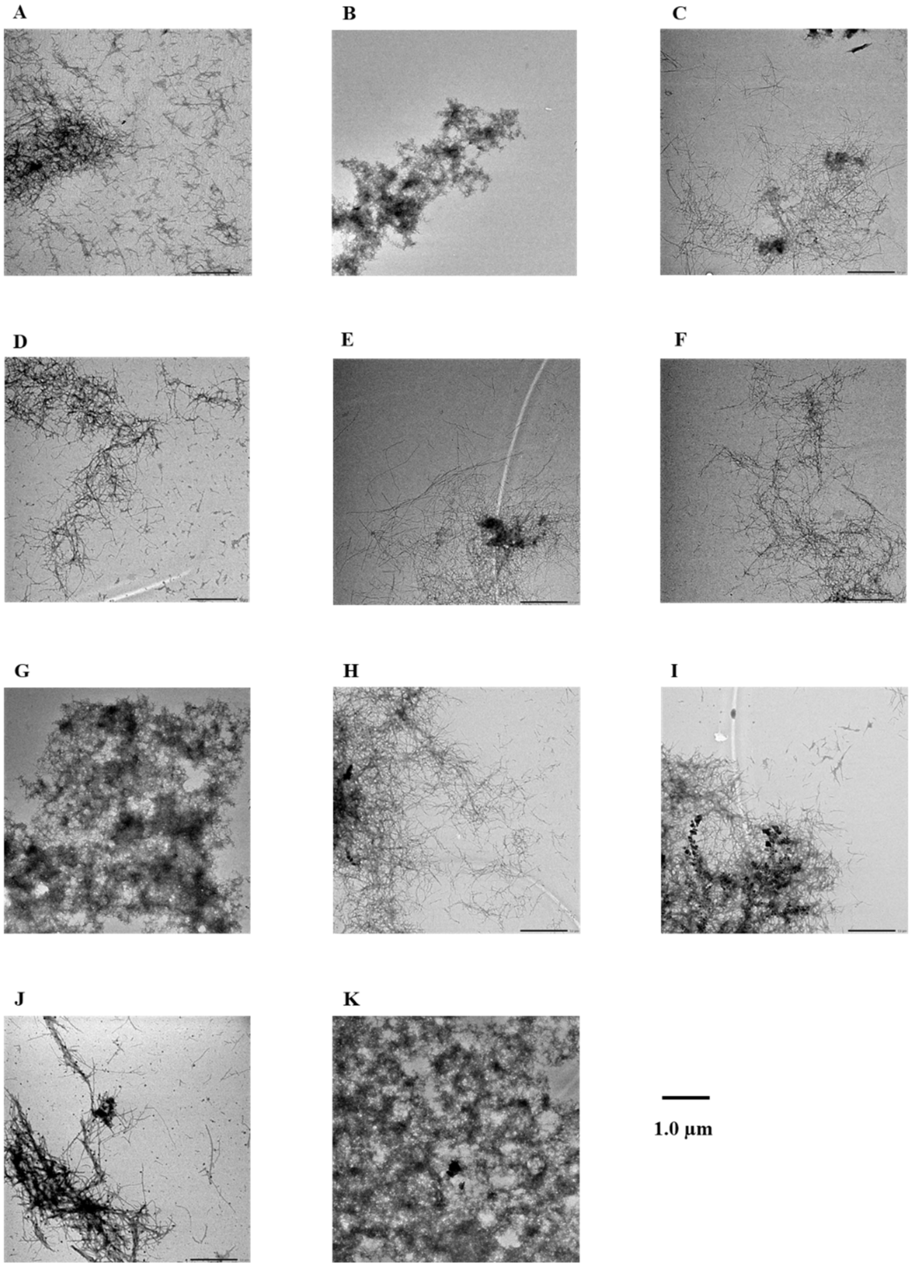

Figure S2

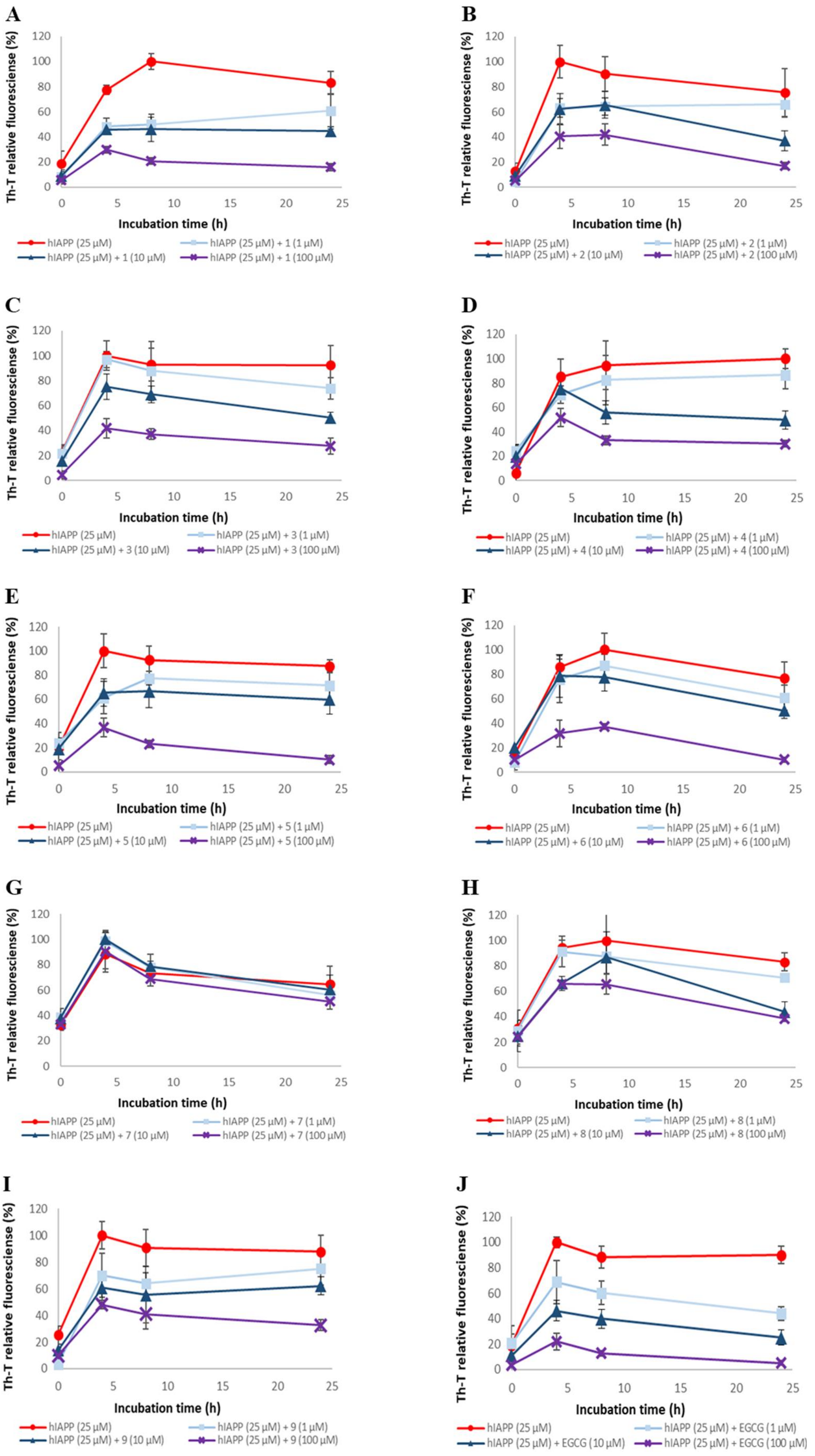
A
Th-T relative fluoresciense (%)
Incubation time (h)
hIAPP (25 μM)
hIAPP (25 μM) + 1 (1 μM)
hIAPP (25 μM) + 1 (10 μM)
hIAPP (25 μM) + 1 (100 μM)
B
Th-T relative fluoresciense (%)
Incubation time (h)
hIAPP (25 μM)
hIAPP (25 μM) + 2 (1 μM)
hIAPP (25 μM) + 2 (10 μM)
hIAPP (25 μM) + 2 (100 μM)
C
Th-T relative fluoresciense (%)
Incubation time (h)
hIAPP (25 μM)
hIAPP (25 μM) + 3 (1 μM)
hIAPP (25 μM) + 3 (10 μM)
hIAPP (25 μM) + 3 (100 μM)
D
Th-T relative fluoresciense (%)
Incubation time (h)
hIAPP (25 μM)
hIAPP (25 μM) + 4 (1 μM)
hIAPP (25 μM) + 4 (10 μM)
hIAPP (25 μM) + 4 (100 μM)
E
Th-T relative fluoresciense (%)
Incubation time (h)
hIAPP (25 μM)
hIAPP (25 μM) + 5 (1 μM)
hIAPP (25 μM) + 5 (10 μM)
hIAPP (25 μM) + 5 (100 μM)
F
Th-T relative fluoresciense (%)
Incubation time (h)
hIAPP (25 μM)
hIAPP (25 μM) + 6 (1 μM)
hIAPP (25 μM) + 6 (10 μM)
hIAPP (25 μM) + 6 (100 μM)
G
Th-T relative fluoresciense (%)
Incubation time (h)
hIAPP (25 μM)
hIAPP (25 μM) + 7 (1 μM)
hIAPP (25 μM) + 7 (10 μM)
hIAPP (25 μM) + 7 (100 μM)
H
Th-T relative fluoresciense (%)
Incubation time (h)
hIAPP (25 μM)
hIAPP (25 μM) + 8 (1 μM)
hIAPP (25 μM) + 8 (10 μM)
hIAPP (25 μM) + 8 (100 μM)
I
Th-T relative fluoresciense (%)
Incubation time (h)
hIAPP (25 μM)
hIAPP (25 μM) + 9 (1 μM)
hIAPP (25 μM) + 9 (10 μM)
hIAPP (25 μM) + 9 (100 μM)
J
Th-T relative fluoresciense (%)
Incubation time (h)
hIAPP (25 μM)
hIAPP (25 μM) + EGCG (1 μM)
hIAPP (25 μM) + EGCG (10 μM)
hIAPP (25 μM) + EGCG (100 μM)

Figure S3

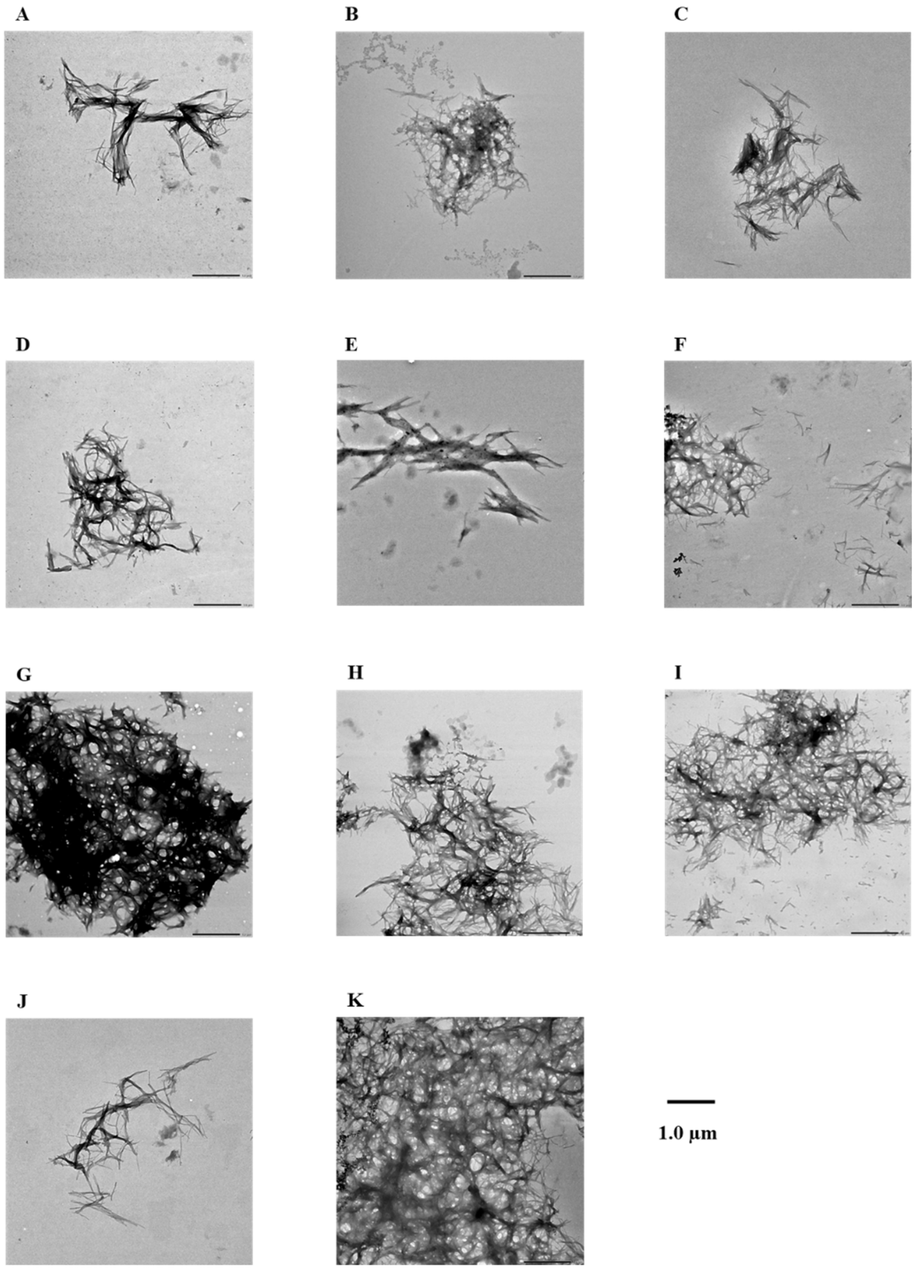

Figure S4

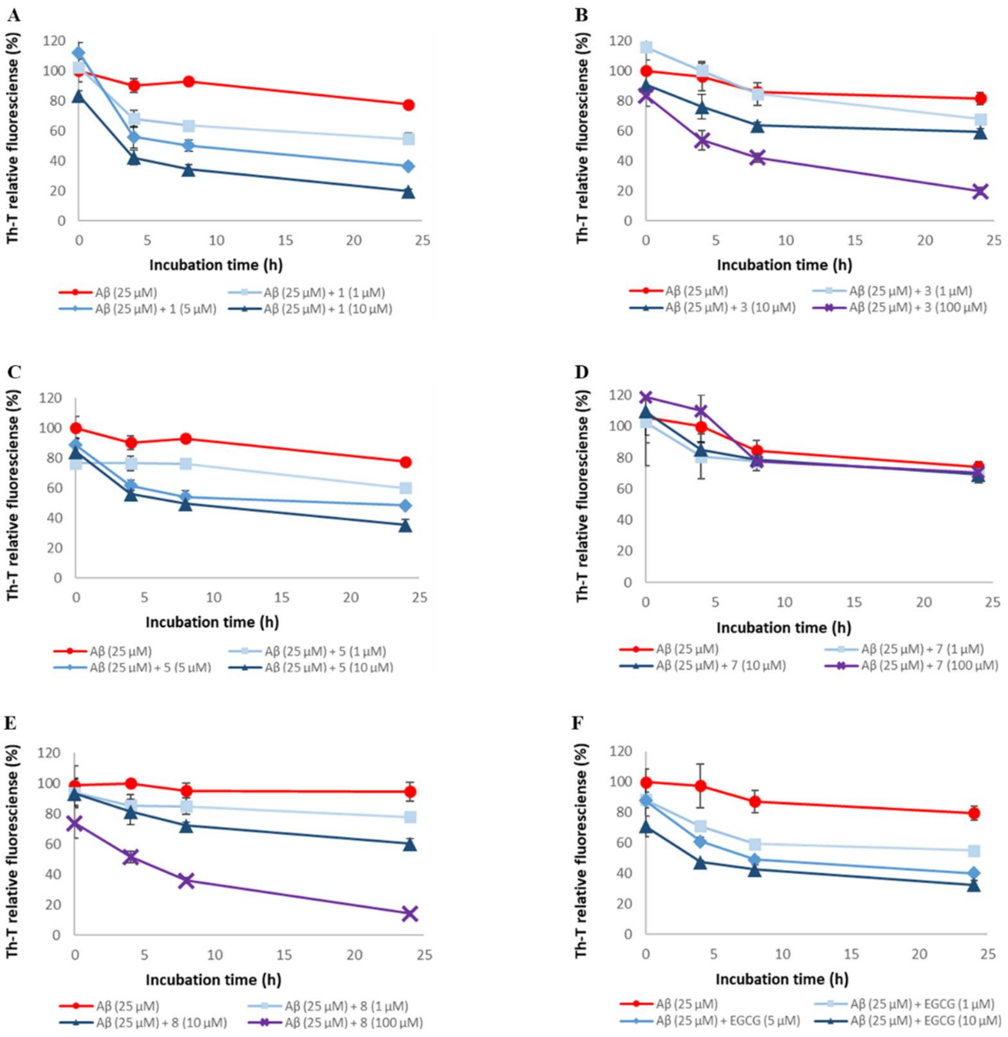

Figure S5

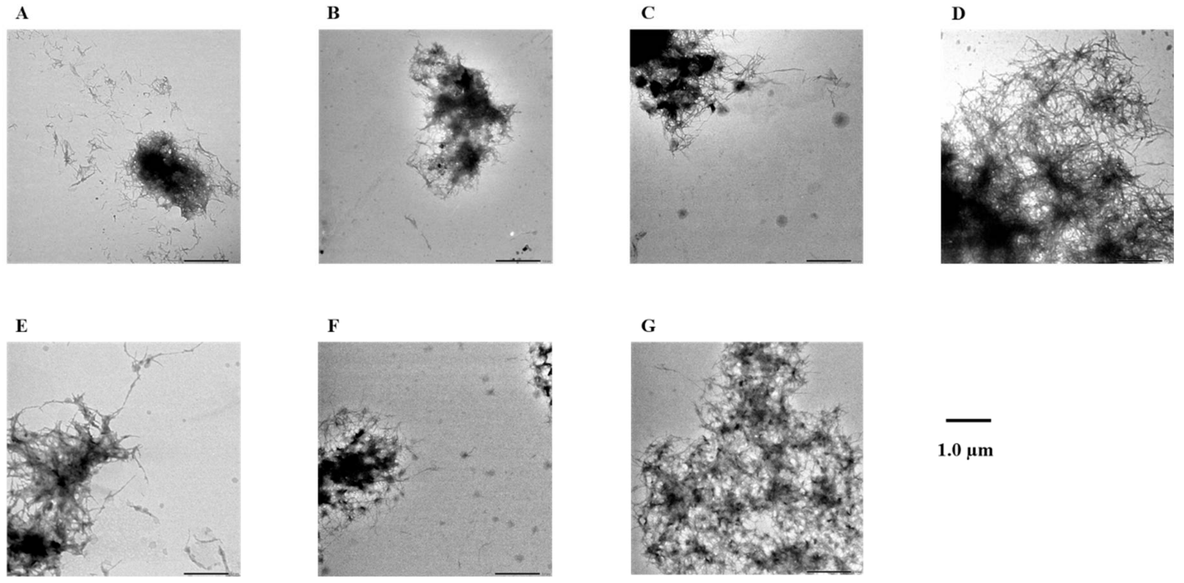

Figure S6

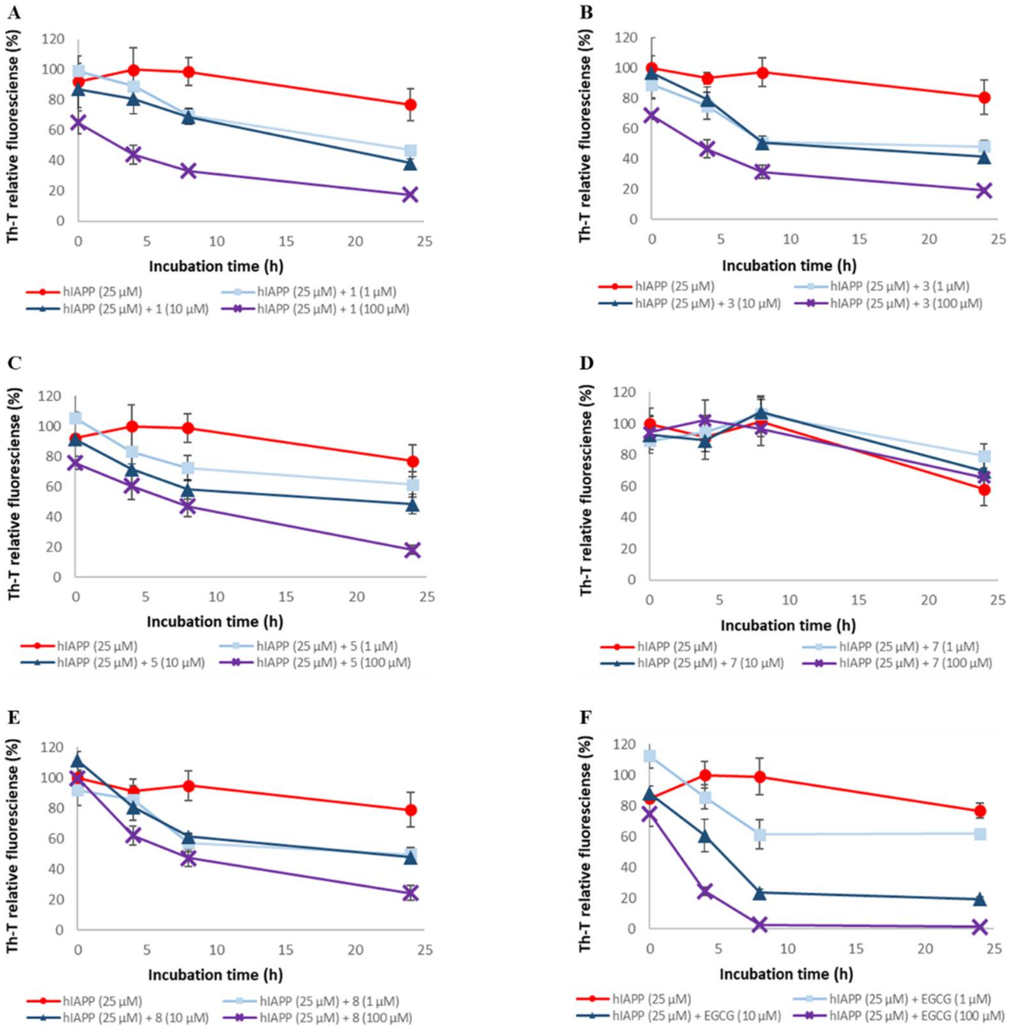

Figure S7

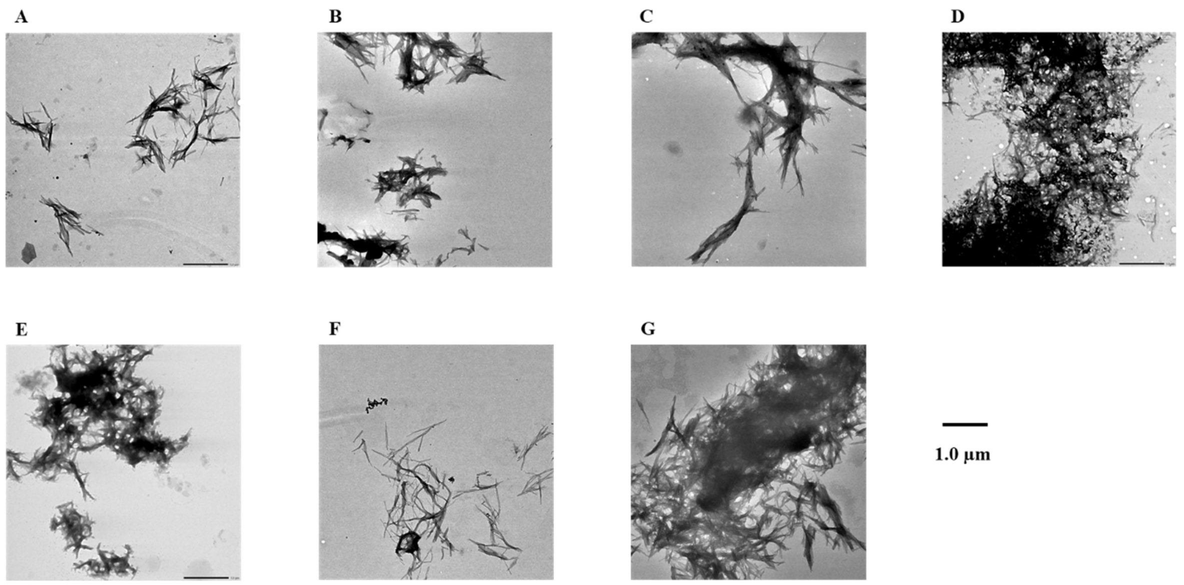

Figure S8
